# CHF6001 Inhibits NF-κB Activation and Neutrophilic Recruitment in LPS-Induced Lung Inflammation in Mice

**DOI:** 10.3389/fphar.2019.01337

**Published:** 2019-11-12

**Authors:** Fabio F. Stellari, Angelo Sala, Francesca Ruscitti, Carola Buccellati, Andrew Allen, Patrizia Risé, Maurizio Civelli, Gino Villetti

**Affiliations:** ^1^Pharmacology and Toxicology Department Corporate Pre-Clinical R&D, Chiesi Farmaceutici S.p.A, Parma, Italy; ^2^Department of Pharmaceutical Sciences, School of Drug Sciences, University of Milan, Milan, Italy; ^3^IBIM, Consiglio Nazionale delle Ricerche, Palermo, Italy

**Keywords:** LPS, inhalation towers, NF-kB, *in vivo* bioluminescence imaging, lung inflammation, mouse model, PDE4

## Abstract

Inhibitors of phosphodiesterase 4 (PDE4) are potent anti-inflammatory agents, inhibiting the production of inflammatory mediators through the elevation of intracellular cAMP concentrations. We studied the activity of a novel PDE4 inhibitor, CHF6001, both *in vitro* in human cells and *in vivo*, using bioluminescence imaging (BLI) in mice lung inflammation. Mice transiently transfected with the luciferase gene under the control of an NF-κB responsive element (NF-κB-luc) have been used to assess the *in vivo* anti-inflammatory activity of CHF6001 in lipopolysaccharide (LPS)-induced lung inflammation. BLI as well as inflammatory cells and the concentrations of pro-inflammatory cytokines were monitored in bronchoalveolar lavage fluids (BALF) while testing *in vitro* its ability to affect the production of leukotriene B_4_ (LTB_4_), measured by LC/MS/MS, by LPS/LPS/*N*-formyl--methionyl--leucyl-phenylalanine (fMLP)-activated human blood. CHF6001 inhibited the production of LTB_4_ in LPS/fMLP-activated human blood at sub-nanomolar concentrations. LPS-induced an increase of BLI signal in NF-κB-luc mice, and CHF6001 administered by dry powder inhalation decreased in parallel luciferase signal, cell airway infiltration, and pro-inflammatory cytokine concentrations in BALF. The results obtained provide *in vitro* and *in vivo* evidence of the anti-inflammatory activity of the potent PDE4 inhibitor CHF6001, showing that with a topical administration that closely mimics inhalation in humans, it efficiently disrupts the NF-κB activation associated with LPS challenge, an effect that may be relevant for the prevention of exacerbation episodes in chronic obstructive pulmonary disease subjects.

## Background

Cyclic AMP (cAMP) represents a critical intracellular second messenger in immune cells such as macrophages (Peters-Golden, 2009) and neutrophils (Bloemen et al., 1997), where it exerts an inhibitory control over activation pathways leading to cytokine production and inflammation. Degradation of cAMP (and cGMP) into inactive products is carried out by phosphodiesterases (PDEs); among the different enzymes belonging to this superfamily, phosphodiesterase 4 (PDE4) represents the cAMP selective isoform mainly expressed in inflammatory and immune cells (Houslay, 2010). Selective inhibition of PDE4 has been predicted to have potential anti-inflammatory activity in lung inflammatory pathologies, such as asthma and chronic obstructive pulmonary disease (COPD), but early on inhaled corticosteroids (ICS) become the anti-inflammatory of choice in asthma, while the use of oral PDE4 inhibitors was complicated by variable activity and the presence of significant side effects, in particular gastrointestinal. In light of these limitations, only one oral PDE4 inhibitor (namely roflumilast) is at present commercially available for use in COPD (Giembycz and Field, 2010), a pathology where at variance with asthma, ICS cannot effectively control airway phlogosis.

CHF6001 is a PDE4 inhibitor optimized for inhaled delivery with the goal of achieving maximal efficacy in airways while reducing systemic exposure and side effects (Moretto et al., 2015; Villetti et al., 2015). When administered by dry powder inhalation, CHF6001 is well tolerated up to 4,800 µg in humans (Mariotti et al., 2018) and is currently in phase IIb clinical trials for the treatment of COPD (clinicaltrials.gov).

LTB_4_ is a potent chemoattractant and activator of leukocytes that significantly enhances neutrophil adhesion to endothelial cells (Hoover et al., 1984), promoting neutrophil infiltration into inflamed tissues (Kim et al., 2006). Its synthesis relies on the availability of arachidonic acid released from membrane phospholipids by the cytosolic phospholipase A2 (cPLA2), as cPLA_2_-null mice cannot produce LTB_4_ (Bonventre et al., 1997). LTB_4_, like other chemoattractants, inhibits activated adenylyl cyclase activity through G_i_-like G-proteins and enhances the activity of nuclear factor kappaB (NF-κB) (Sanchez-Galan et al., 2009; Serezani et al., 2011).

NF-κB is a redox-sensitive transcription factor that plays a critical role in a wide array of inflammatory networks regulating cytokine production in airway pathologies, including COPD (Schuliga, 2015). NF-κB activation in pulmonary inflammation is mainly induced by mediators such as interleukin (IL)-1β, tumor necrosis factor (TNF)-α, or by bacterial or viral exacerbations activating Toll-like receptors (Edwards et al., 2009). NF-κB may also be involved in the control of five-lipoxygenase activating protein (FLAP, a key protein for leukotriene synthesis in intact cells) expression and may therefore increase LTB_4_ production in a vicious positive feedback cycle (Gonsalves and Kalra, 2010). Modulation of NF-κB activation using both small molecules, decoy oligonucleotides, or siRNA has been successful in animal models, but transition to humans still requires the identification of specific targets and the development of active inhibitors (Rahman and Fazal, 2011).

In the present study, we evaluated the anti-inflammatory activity of CHF6001 both *in vitro* and *in vivo*, assessing the ability to inhibit the production of the potent neutrophil chemotactic factor LTB_4_ in lipopolysaccharide (LPS)/*N*-formyl--methionyl--leucyl-phenylalanine (fMLP)-activated human blood and the effects on the pulmonary inflammatory response, resulting from LPS intratracheal challenge. We assessed the anti-inflammatory activity *in vivo* administering the dry powder developed for clinical use through an inhalation tower by enabling the testing in animals of the same formulation administered to patients and monitored pulmonary inflammation with the noninvasive assessment of NF-κB activation in mice transiently transfected with the luciferase gene under the control of NF-κB responsive element.

The results obtained with CHF6001 showed a potent inhibition of critical components of the inflammatory response elicited by LPS, especially *in vivo* upon a topical administration that closely mimics inhalation in humans and supports a significant anti-inflammatory activity by CHF6001 that may be fully exploited for the prevention of exacerbation episodes in COPD subjects.

## Methods

### Animals

Female FVB (7–8 weeks old) mice were purchased from Harlan Laboratories Italy (S. Pietro al Natisone, Udine, Italy).

In this study, only female mice were used since male rats and mice were used to evaluate the anti-inflammatory activity of CHF6001 in a study we published previously (Villetti et al., 2015) and we do not expect any difference in LPS challenge between the male and female.

Animals were maintained under conventional housing conditions. Prior to use, animals were acclimated for at least 5 days to the local vivarium conditions (room temperature: 20–24°C; relative humidity: 40–70%), having free access to standard rat chow and tap water. All animal experiments described herein were approved by the intramural animal welfare committee for animal experimentation of Chiesi Farmaceutici under protocol number 0013415-P-25/07/2011 which comply with the European Directive 2010/63 UE, Italian D.Lgs 26/2014, and the revised “Guide for the Care and Use of Laboratory Animals” (Guide for the Care and Use of Laboratory Animals, 1996).

### Reagents

LPS (from *Escherichia coli* 0111:B4, product n.L3012) was from Sigma (St. Louis, MO); JetPEI was from Polyplustransfection Inc (Euroclone, Milano, Italy); NF-κB vector (pGL4.32[luc2P/NF-jB-RE/Hygro]) was from Promega (Madison, WI); CHF6001 (M.W. 687.54) was synthesized at Chiesi Farmaceutici S.p.A., Parma, Italy (Armani et al., 2014). Lactose (Inhalac, containing magnesium stearate only) was used as vehicle, and micronized CHF6001 blend and vehicle were compressed into the canister using a hydraulic press. [d_4_]LTB_4_ and LTB_4_ were from Cayman Chem. (Ann Arbor, MI).

### LTB_4_ Production in Human Whole Blood

LTB_4_ production was elicited in human blood cells according to Brideau et al. (1999), with modifications. Briefly, human “buffy coat” (Blood Bank, University of Milan) diluted 1:4 with homologous plasma and 10 UI/ml heparin was treated with LPS (1 µg/ml) at 37°C with or without CHF6001 (0.1 pM–1 µM), the FLAP inhibitor MK886 (1 µM), the selective COX2 inhibitor lumiracoxib (30 nM), or the non-selective COX1–COX2 inhibitor indomethacin (30 µM). After 24 h, samples were added with LPS again for 30 min followed by the formylated tripeptide fMLP (1 µM) for additional 15 min at 37°C. Supernatants from rapid centrifugation at 13,000 × *g* for 3 min, 4°C were recovered, added with three volumes of acetonitrile containing [d_4_]LTB_4_, and maintained at −20°C until analysis.

Each treatment had two replicates with cells from the same donor and concentration–response curves were originated using the average values from at least three different donors.

### LTB_4_ Analysis by LC/MS/MS Mass Spectrometry

Supernatants from blood incubations, containing the deuterated internal standard for the stable isotope dilution quantitative determination, were centrifuged at 13,000 × *g* for 5 min, 4°C to precipitate proteins and, upon dilution with two volumes of solvent A (ammonium acetate buffer, pH 5.7), analyzed by LC/MS/MS using selected reaction monitoring of the specific transitions from *m*/*z* 335 to 195 (LTB_4_) and *m*/*z* 339 to 197 ([d_4_]LTB_4_) as previously described (Zarini et al., 2009).

Quantitation was obtained using standard curves of synthetic LTB_4_.

### Vector Characteristic and *In Vivo* Gene Delivery

The pGL4.32[luc2P/NF-jB-RE/Hygro] vector (GenBank/EMBL accession number EU581860) contains five copies of an NF-κB responsive element (NF-κB-RE) that drives transcription of the luciferase reporter gene luc2P (*Photinus pyralis*). Multimerization of responsive elements is frequently used to increase the sensitivity to a specific transcription factor. In this case, five copies of NF-kB have been considered a good compromise to increase sensitivity and maintain physiological conditions. Higher copy numbers of a NF-kB responsive element could lead to recruit unspecific transcription factors, dysregulating the promoter activation.

Luc2P is a synthetically derived luciferase sequence with humanized codon optimization that is designed for high expression and reduced anomalous transcription. The luc2P gene contains hPEST, a protein destabilization sequence. The protein encoded by luc2P responds more quickly than the protein encoded by the luc2 gene upon induction. The vector backbone contains a resistance gene to allow selection in *E. coli* and a mammalian selectable marker for hygromycin resistance. JetPEI (Wu et al., 2004; Oh et al., 2013) was applied *in vivo* as a carrier for delivering DNA to lung tissues. The DNA and JetPEI were formulated according to the product manual. Briefly, 40 µg of NF-κB-luc reporter and 7 µl of JetPEI were each diluted into 100 µl of 5% glucose. The two solutions were then mixed and incubated for 15 min at room temperature. The entire mixture (approximately 200 µl) was injected into the tail vein of mice. Both DNA and JetPEI are toxic if injected at high doses, and therefore a careful optimization of the transfection conditions also in terms of DNA and JetPEI relative ratio has been performed to obtain the desired lung transfection efficiency and avoid or minimize *in vivo* toxicity. The intravenous route of administration has been selected based on our preview studies showing that upon intravenous injection, *in vivo* JetPEI-mediated DNA delivery leads to gene expression mainly in the lung ((Stellari et al., 2012; Stellari et al., 2014a; Stellari et al., 2015a; Stellari et al., 2017).

The optimized transfection protocol, as described, has been applied also in this study, and, following LPS challenge, we have observed a fold induction of bioluminescence in lung tissue comparable with our previous studies. Suggestions regarding different ways of administration and protocol for *in vivo* gene delivery have been proposed by PolyPLus (https://www.polyplus-transfection.com).

### *In Vivo* Bioluminescence Imaging

Bioluminescence imaging (BLI) is a noninvasive method, and therefore the 3R rule is intrinsic to its nature.

As previously reported (Stellari et al., 2014b), transfection *per se* causes a mild lung inflammatory response and NF-κB activation that is detectable by BLI up to 3–4 days after DNA injection and disappears completely after 1 week. Therefore, 1 week after DNA delivery, the transient transgenic mice were injected with luciferin i.p. and BLI was recorded. Briefly, following intraperitoneal injection of luciferin (150 mg/kg), mice were lightly anesthetized with isoflurane (2.5%) and images were obtained using an IVIS imaging system (PerkinElmer, Inc., Waltham, MA) at 10 and 15 min after luciferin: an average of photons emitted from the chest of the mice was quantified using Living ImageJ software (PerkinElmer, Inc., Waltham, MA). The following day, mice were intratracheally challenged with LPS (12.5 µg/mouse) and BLI was recorded at 2, 4, 7, and 24 h after LPS instillation, 15 min after i.p. injection of luciferin (150 mg/kg).

### Acute Pulmonary Inflammation: Effect of CHF6001

Intratracheal (i.tr.) challenge with LPS was carried out using 50 µl of LPS solution [250 µg/ml in phosphate-buffered solution (PBS)] and a 22-gauge intubator, resulting in a final dose of LPS of 12.5 µg/mouse, with the control group receiving 50 µl of saline i.tr.

The mice were treated by the snout-only inhalation route as a 15-min exposure. Test groups were exposed to an atmosphere containing CHF6001 (supplied as a nominal 4% (*w*/*w*) blend in vehicle). Different doses were achieved by varying the concentration of the CHF6001 in the tower. The control group animals were exposed to the vehicle.

At least one sample was collected from each exposure system.

Concentration samples used an open-face filter and particle size analysis was carried out using a Marple (model 296) Personal Cascade Impactor with stainless steel collection substrates and a glass fiber filter. Samples were collected from each test group. The mass median aerodynamic diameter (MMAD) and geometric standard deviation of the aerosol were derived for each occasion of measurement.

Collected samples were retained for analysis of CHF6001 by LC/MS/MS (Villetti et al., 2015).

### Broncho-Alveolar Lavage Fluid Analysis

Bronchoalveolar lavage (BAL) fluid collection was performed 24 h after LPS challenge. Mice were sacrificed by an overdose of isoflurane and a midline neck incision was performed to cannulate the trachea. Lungs were washed three times with 0.6 ml of PBS as previously described (Nassini et al., 2012). Cell counts were performed with an automated cell counter (Sysmex XT100V, Dasit).

For flow cytometric analysis, cells were labeled in PBS (Euroclone) and 0.5% bovine serum albumin (BSA, Milteny Biotech) with fluorochrome-labeled monoclonal antibodies: anti-mouse CD 45 PE-Cy5 (BD Pharmigen), anti-mouse F4/80 Alexa 488 (AbD Serotec), anti-mouse Lys6G (BD Pharmigen), anti-mouse CD11b PE-Cy7 (BD Pharmigen), and appropriate isotype controls for 30 min at RT in the dark. Cells were washed before and after the staining and resuspended in 300 µl of PBS/BSA. Samples were collected on a FACS Canto II (two lasers, six colors, Becton Dickinson) and analyzed using Diva 7 software. Mean fluorescence intensity (MFI) was determined on a statistically significant number of cells each sample. To positively select all leukocytes in BAL and discard debris, gating was performed on CD45-positive cells. Anti-mouse F4/80 was used to discriminate granulocyte population, including eosinophils and neutrophils, from macrophages. Lymphocytes were gated out based on forward scatter (FSC) and side scatter (SSC). Moreover, anti-mouse GR1^+^ was used to negatively gate out all neutrophils. Fluorescence-activated cell sorting (FACS) analysis finally quantitated CD11b surface activation marker expression on the remaining population of monocytes/macrophages characterized as CD45^+^ F4/80^+^ GR1^−^ cells.

Cytokine and chemokine levels were measured in the bronchoalveolar lavage fluid (BALF) supernatants using a Bio-Plex™ Cytokine Assay Kit (Bio-Rad Laboratories, Segrate, Milano, Italy) according to the manufacturer’s instructions as previously published (Stellari et al., 2014b; Stellari et al., 2015a).

### Statistical Analysis

As tests for normality were positive, statistical analysis was performed on raw data using one-way analysis of variance (ANOVA) followed by Dunnett’s *t post hoc* test for comparison with control groups. Experimental values were expressed as the mean and standard error of the mean (SEM) of *n* observations. (*P < 0.05, **P < 0.01, ***P < 0.001). Sigmoidal dose–response curve was obtained and IC_50_ calculated using GraphPad Prism 4.00. 

## Results

### Effect of CHF6001on LTB_4_ Production by LPS-Activated Human Blood Cells

**Figure 1:**
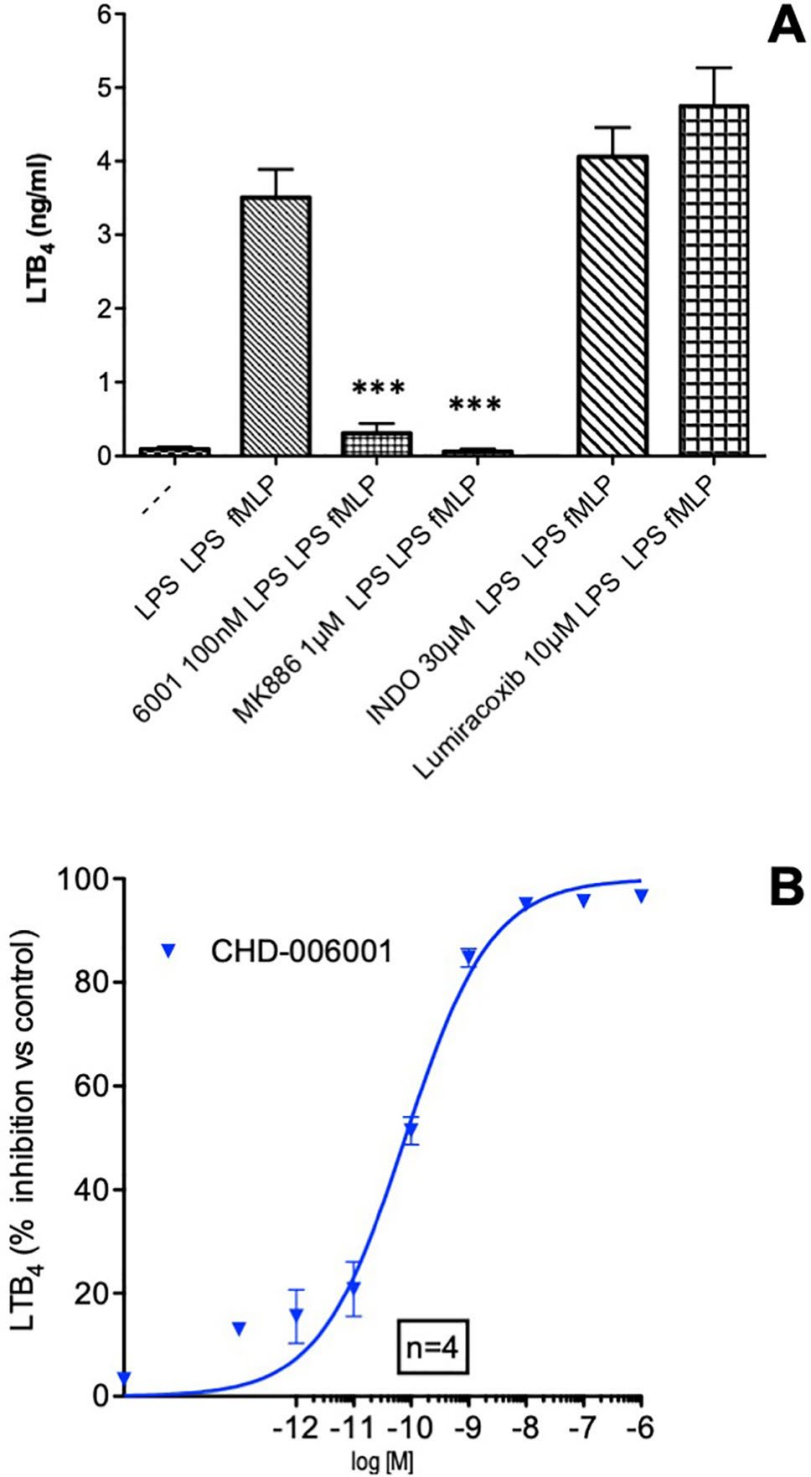
LTB_4_ inhibition by CHF6001. **(A)** Human blood was treated with LPS (1 µg/ml) in the presence or absence of different compounds, followed by lipopolysaccharide (LPS) again after 24 h and the formylated tripeptide *N*-formyl--methionyl--leucyl-phenylalanine (fMLP, 1 µM) as described in *METHODS*. LTB_4_ production was assessed by LC/MS/MS using stable isotope dilution. **(B)** Concentration–response curve of the inhibitory effect of CHF6001 on LTB_4_ production by LPS/LPS/fMLP activated human blood, assessed by LC/MS/MS using stable isotope dilution. Values are shown as the mean ± SEM, *n* = 4 for each group. ***P < 0.001 *versus* LPS group (Dunnett’s test). Sigmoidal dose–response curve was obtained using GraphPad Prism 4.00.

The 24-h incubation with the bacterial membranes LPS followed by LPS and fMLP activation resulted in a significant production of LTB_4_, in agreement with previous reports (Brideau et al., 1999). Synthesis of LTB_4_ was completely prevented in the presence of the leukotriene synthesis inhibitor MK886 (1 µM), a compound preventing docking of 5-lipoxygenase to the nuclear membrane (Dixon et al., 1990) and access to the substrate arachidonic acid released by phospholipase A_2_ (Abramovitz et al., 1993), but was unaffected by either the specific COX-2 inhibitor lumiracoxib or the nonspecific COX1–COX2 inhibitor indomethacin (**Figure 1A**). CHF6001 concentration-dependently prevented the formation of the potent neutrophilic chemotactic factor LTB_4_ with an IC_50_ of 79 pM (**Figure 1B**), but was ineffective against direct activation of 5-LO by the calcium ionophore A23187 (data not shown).

### Inhaled Dose of CHF6001

**Figure 2:**
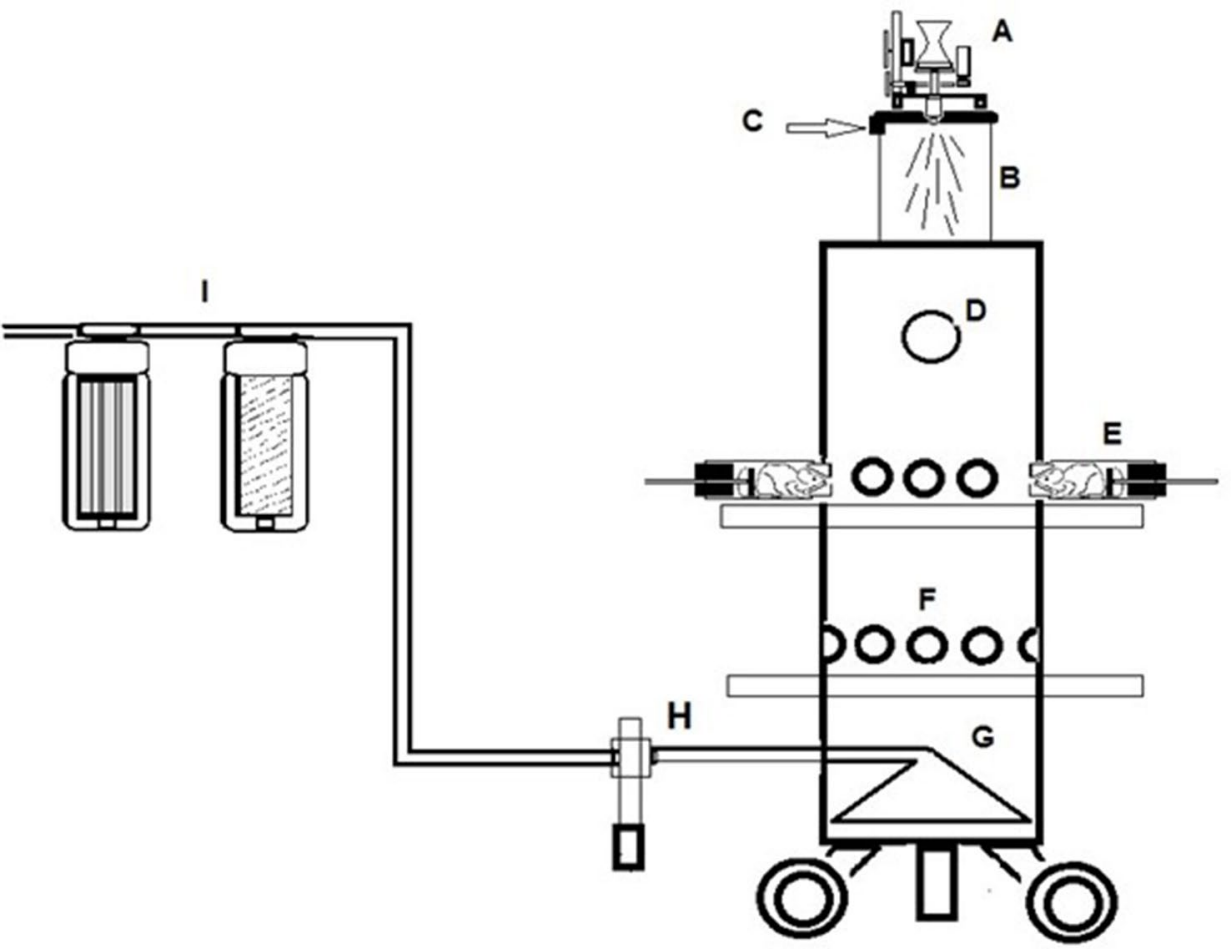
A schematic of the Chiesi inhalation towers. **(A)** Wrights Dust Feed. **(B)** Pre-chamber. **(C)** Tangential air inlet. **(D)** Viewing port in top section. **(E)** Restraint tube attached to the tower. **(F)** Vacant animal ports closed off with bungs. **(G)** Conical exhaust plenum. **(H)** Aerosol monitoring device. **(I)** Filtration units in extract.

Monitoring aerosol concentrations of CHF6001 dry powder delivered within the airways of the experimental animals using the snout-only delivery tower (**Figure 2**) resulted in an estimated dose inhaled close to target at all three doses used and showed good inter-experiment reproducibility (**Table 1** and **Table 2)**.

**Table 1:** Estimated inhaled doses (mg/kg) of CHF6001.

Target dose (mg/kg)	Estimated inhaled dose (mean of 2 doses)	% of Target
0.1	0.0856	85.6
0.3	0.290	96.7
0.6	0.664	111
0.6 repeat	0.631	105

The inhaled dose was derived using the following equation [43]: $\text{Delivered dose}(\mathrm{\mu g}/\mathrm{kg})=\frac{\mathrm{Conc}\times \mathrm{RMV}\times D}{\mathrm{BW}}$

Where Conc., concentration of CHF6001 in air inhaled (µg/l).

RMV, volume of air inhaled in 1 min (l/min) 0.608 x BW (kg)^0.852^.

D, duration of exposure to the aerosol (min).

BW, body weight (kg).

**Table 2:** Mass median aerodynamic diameter (MMAD, µm) of CHF6001.

CHF6001 dose (mg/kg)	MMAD (µm)	*σg*
0.1	1.7	2.19
0.3	1.9	2.25
0.6	2.1	2.23

MMAD, Mass median aerodynamic diameter.

σg, Geometric standard deviation.

### Effect of CHF6001 on Acute Pulmonary Inflammation in Mice

**Figure 3:**
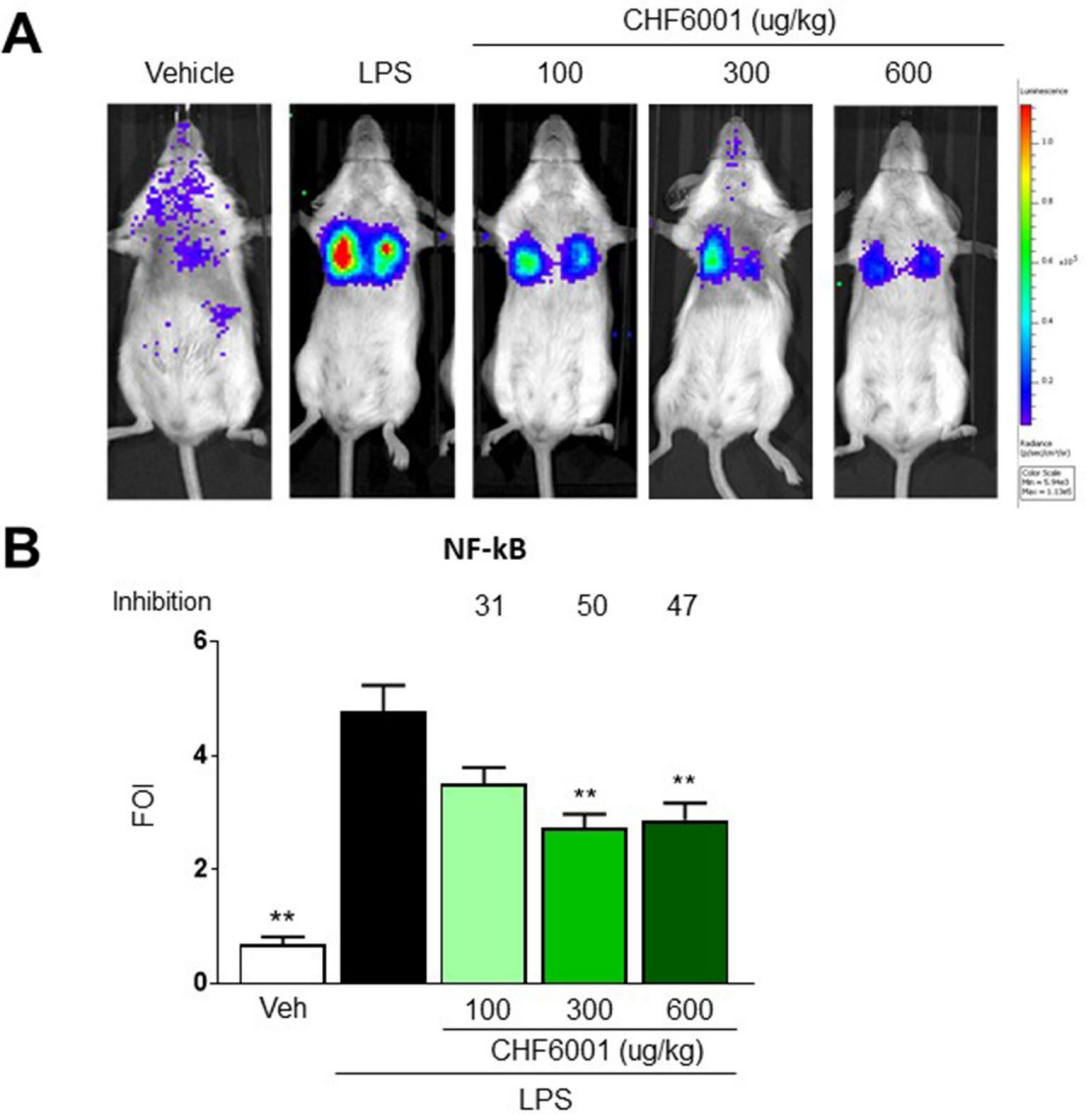
Effects of CHF6001 on NF-kB activation *in vivo* as assessed by bioluminescence imaging (BLI). **(A)** Transient NF-kB-luc transgenic mice were pretreated with saline or CHF6001 (100–600 µg/kg by inhalation) 1 h before lipopolysaccharide (LPS) intratracheal instillation. Representative mice are shown for saline, LPS, LPS+CHF6001 (100 µg/kg), and LPS+CHF6001 (600 µg/kg). **(B)** Quantification of NF-kB activation by BLI, 4 h after LPS intratracheal instillation. Values are shown as the mean ± SEM, *n* = 6, and percent inhibition *versus* LPS group is reported. **P < 0.01 *versus* LPS group (Dunnett’s test).

As previously reported (Ansaldi et al., 2011; Stellari et al., 2014b; Stellari et al., 2015b), LPS intratracheal instillation 1 week after DNA delivery of the luciferase reporter construct caused the activation of NF-kB in the lungs, which could be easily monitored by BLI showing a very marked increase when compared to control animals. Luciferase expression driven by NF-kB activation was detectable by BLI as early as 2 h after LPS challenge and peaked at 4 h after treatment, with a fivefold induction over baseline (**Figures 3A and B**). The signal was still significantly enhanced 7 h after LPS, but returned to baseline levels 24 h after LPS challenge (data not shown).

Pretreatment with CHF6001 by Inhalation using the snout-only delivery tower, closely mimicking inhalation in humans, dose-dependently reduced the BLI Signal (**Figure 3A**). quantitative determination of bli in the transient transgenic mice showed a maximal inhibition of the increase in BLI caused by LPS of ≈50% (**Figure 3B**).

**Figure 4:**
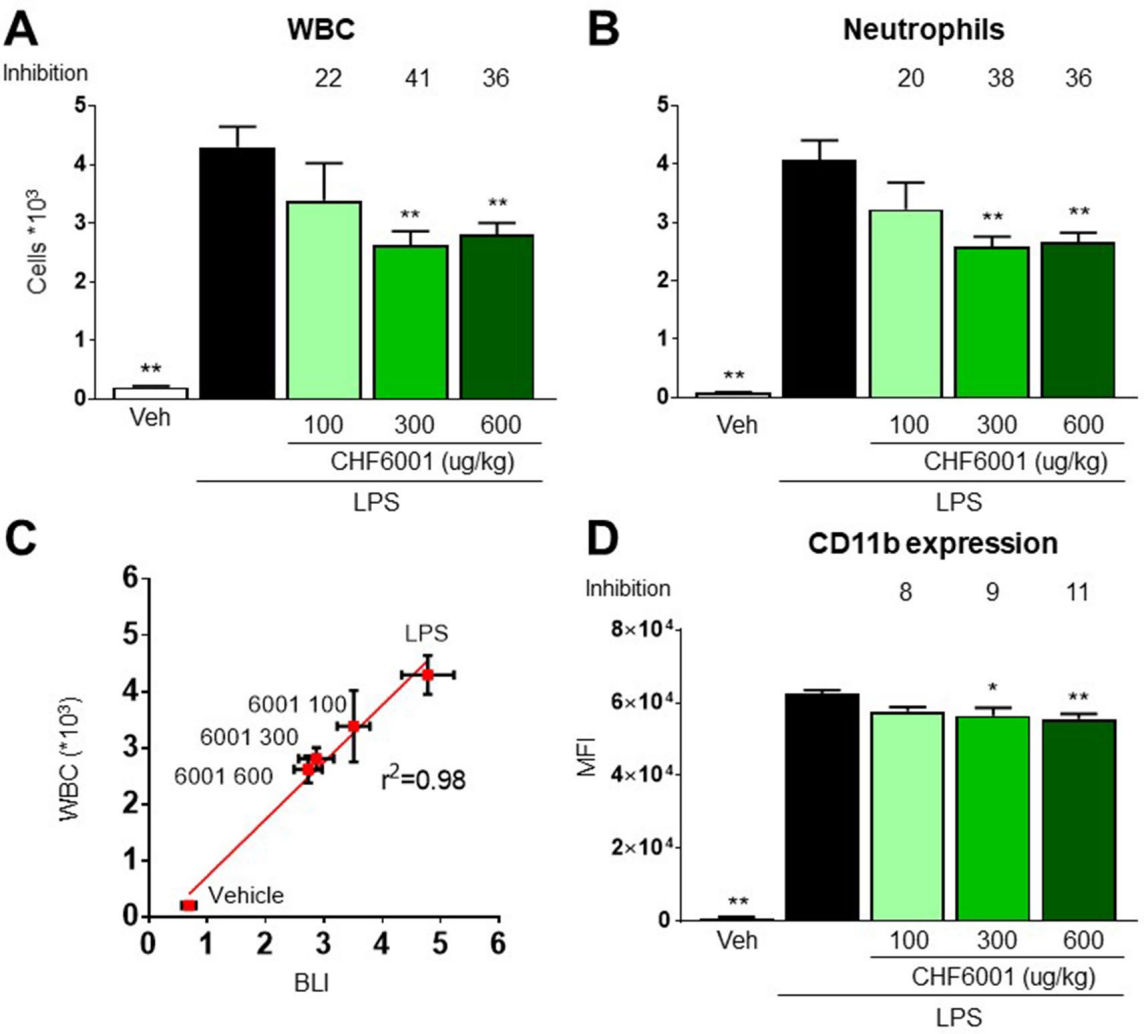
Effects of CHF6001 on lipopolysaccharide (LPS)-induced neutrophils and white blood cell (WBC) recruitment and on monocyte/macrophages adhesion molecule expression. **(A)** and **(B)** Transient NF-kBluc transgenic mice were pretreated with saline or CHF6001 (100–600 µg/kg) 1 h before LPS and sacrificed 24 h after LPS administration by tracheal instillation. WBC **(A)** and neutrophils **(B)** were counted with an automated cell counter. **(C)** Correlation between NF-kB activation as measured by BLI, 4 h after LPS instillation, and the concentrations of WBC in bronchoalveolar lavage fluids, obtained 24 h after LPS instillation. **(D)** CD11b surface activation marker expression on monocytes/macrophages characterized as CD45^+^ F4/80^+^ GR1^−^ cells. Values are shown as the mean ± SEM, *n* = 6 for each group, and percent inhibition *versus* LPS group is reported. *P < 0.05, **P < 0.01 *versus* LPS group (Dunnett’s test).

The numbers of white blood cells (WBC) and neutrophils recovered by BAL 24 h after LPS challenge increased by over 20-/40-fold when compared to vehicle-treated animals, and this increase was also dose-dependently inhibited with CHF6001 inhalation pretreatment by 41%/38% (**Figures 4A and B**). A highly statistically significant correlation was observed between BLI quantified 4 h after LPS administration and the number of WBC and neutrophils recovered by BAL 24 h after LPS challenge (**Figure 4C**), strongly supporting the presence of a quantitative correlation between NF-kB activation, BLI determination, and the immune response represented by white blood cell infiltration of the lung airways. The marked inhibition of BLI/WBC infiltration confirmed the potent pulmonary anti-inflammatory activity of the compound administered by inhalation.

LPS-driven NF-kB activation also contributes to neutrophil extravasation and infiltration by inducing the expression of adhesion molecules (Zhou et al., 2005), and CD11b expression analysis by FACS on neutrophils recovered in BAL reported a very marked increase following LPS challenge, an increase that was also dose-dependently affected by pretreatment with CHF6001 (**Figure 4D**).

**Figure 5:**
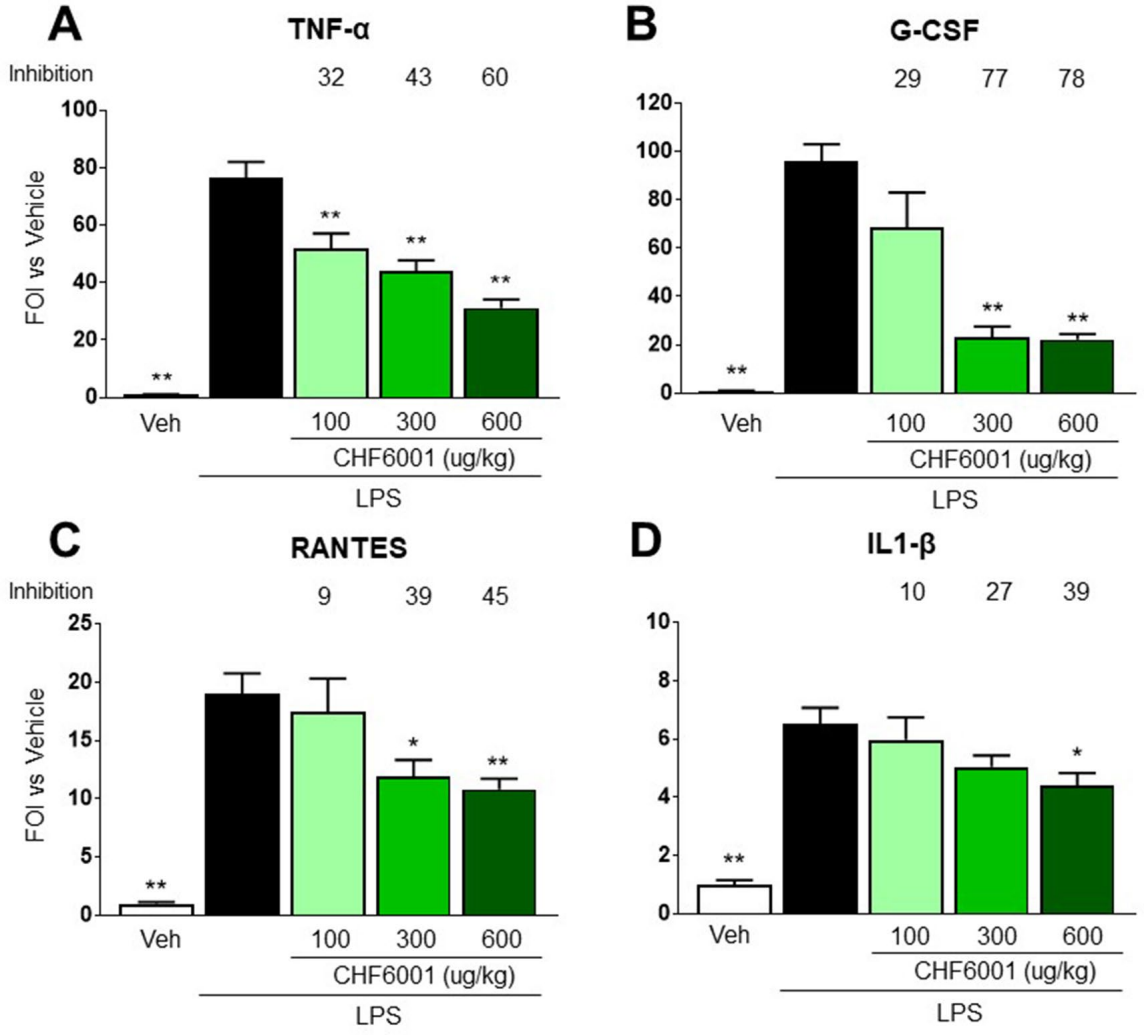
Effects of CHF6001 on cytokine concentrations in bronchoalveolar lavage fluids. Transient NF-kB-luc transgenic mice were pretreated with saline or CHF6001 (100–600 µg/kg) 1 h prior to lipopolysaccharide (LPS) and sacrificed 24 h after tracheal instillation of LPS. Cytokines were assessed using a Bio-Plex™ Cytokine Assay Kit as described in *METHODS*. **(A)** Tumor necrosis factor alpha (TNF-α). **(B)** Granulocyte colony-stimulating factor (G-CSF). **(C)** Regulated on activation, normal T cell expressed and secreted (RANTES). **(D)** IL-1β. Values are shown as the mean ± SEM, *n* = 6 for each group. *P < 0.05, **P <0.01 *versus* LPS group (Dunnett’s test).

In a similar fashion, LPS-induced increase in BAL concentrations of several pro-inflammatory cytokines, such as TNF-α, granulocyte colony-stimulating factor (G-CSF), interleukin 1β (IL-1β), and regulated on activation, normal T cell expressed and secreted (RANTES) (**Figures 5A–D**), was markedly inhibited by CHF6001, underscoring its effective anti-inflammatory activity.

## Discussion

Increase in airway inflammation is a critical feature of exacerbations in COPD subjects and patients with frequent exacerbations are showing increased mortality rates (Soler-Cataluña et al., 2005). Several factors such as infections, increased oxidative stress, and altered immunological response may be the cause of such a rise in inflammation during exacerbations. In particular, bacterial infections account for a significant fraction of all exacerbations (White et al., 2003) and have been reported to cause increased airway inflammation; biomarkers of inflammation such as MPO, neutrophil elastase activity, IL-8, and LTB_4_ levels all are positively related to sputum bacterial load in fresh sputum samples from COPD patients (Hill et al., 2000). At the same time, NF-kB activation has been reported in inflammatory cells within the airways of COPD subjects during COPD exacerbations (Caramori et al., 2003; Drost et al., 2005), suggesting a potential role for this transcription factor in the inflammatory burst associated with exacerbations.

In the present paper, we describe the potent anti-inflammatory activity of a novel inhaled PDE4 inhibitor that may represent a step forward in the prevention and management of acute events in COPD patients. CHF6001 has been developed (currently in phase IIb clinical trials, see clinicaltrials.gov) as a new drug specifically designed for inhalation delivery (Moretto et al., 2015), with the goal of limiting systemic exposure and avoid the serious, dose-limiting side effects of oral PDE4 inhibitors such as roflumilast (Page and Spina, 2011; Gupta, 2012; ). In order to closely mimic the inhalatory route of administration used in humans, we used the dry powder developed for clinical use, delivering it through snout-only inhalation towers.

The ability of CHF6001 to suppress LTB_4_ formation by human blood *in vitro*, without directly affecting 5-lipoxygenase, suggests that the inhibition of PDE4 at sub-nanomolar concentrations can effectively lower the overall inflammatory response of blood leukocytes to the immunological stimulus represented by bacterial wall lipopolysaccharides. LTB_4_ has been reported, together with IL-8, among the chemoattractants involved in the recruitment of neutrophils in the airways, and its concentrations are increased in induced sputum from COPD subjects (Profita et al., 2005) and further increase during exacerbations (Biernacki et al., 2003).

The potent *in vitro* inhibition of cytokine and chemokine production from LPS-activated peripheral blood mononuclear cells and macrophages caused by CHF6001 (Moretto et al., 2015) was confirmed *in vivo* in a widely used model of airway acute inflammatory response elicited by the local instillation of bacterial LPS in mice. Preclinical models of COPD are often of limited relevance for the pathology in humans; however, monitoring the inflammatory response using a NF-kB activation reporter gene in a transient transgenic mouse allowed to follow a specific molecular event that plays a critical role at the crossroad of multiple inflammatory triggers.

The inhalation route is increasingly adopted to deliver local or systemic therapeutics, and rodent models used in development and tolerability studies should mimic as closely as possible the intended formulation and delivery methods. Tracheal intubation with insufflation of metered dry powders has been used to deliver directly into the lungs, but the method requires invasive surgery, extensive skills, and has been characterized as resulting in irreproducible dosage delivery, lung damages, and respiratory failure in significant numbers of animals (Diot et al., 2012). In order to fill as much as possible the gap between preclinical models and the real life of human administrations, we used inhalation towers to treat mice with a dry powder formulation of CHF6001 used in clinical trials. Inhaled administration of CHF6001 significantly and dose-dependently turned off NF-kB-dependent luciferase activity in LPS-challenged mice and the ability to monitor NF-kB activation noninvasively by BLI is pertinent to the 3R rule. Screening all mice for the basal level of NF-kb activation before entering the experimental protocol, and using each mouse as its own control, greatly limited the variability and allowed the use of a reduced number of animals.

When compared to LPS-challenged, non-treated animals, the inhibition of LPS-induced NF-kB activation by CHF6001 resulted associated to a significant suppression of lung cellular infiltrates as well as of the concentrations of NF-kB-activating inflammatory cytokines such as IL-1β and TNF-α in BAL at 24 h after challenge.

Expression of endothelial adhesion molecules facilitating cell migration as well as activation of monocyte–macrophages may result from production of TNF-α, which is known to be increased in sputum during exacerbations (White et al., 2003). The inhibition on the expression of adhesion molecules by FACS analysis upon CHF6001 topical treatment, together with the decreased number of infiltrating WBC, is also in line with the observed decrease of TNF-α in BALF concentrations.

Recent evidence in knockout mice suggests that G-CSF may play a previously unreported, relevant pathogenic role in COPD (Tsantikos et al., 2018); CHF6001 marked inhibition of G-CSF production may therefore contribute to the overall control of the inflammatory response elicited by bacterial lipopolysaccharides in our model as well as during infection-driven exacerbations in COPD subjects.

The overall ability of inhaled CHF6001 to hamper the production of a number of relevant pro-inflammatory mediators, including RANTES and IL-1β, suggests that this compound may represent a significant step forward in the search for an improved control of acute airway inflammation associated with bacterial infections in COPD. Taken together, these results bode well for the development of much needed therapeutics for the treatment of COPD. The combination of an advanced delivery route and *in vivo* imaging techniques can therefore be used in support of undergoing clinical trials to either identify biomarkers that can be used (activation of NF-kB, production of LTB_4_, TNF-β, G-CSF, etc.) or direct the enrolment of patients with specific phenotypes (such as subjects with a high bacterial load, given the observed efficacy in modulating the inflammatory reaction associated to the exposure to a component of Gram-negative bacterial wall).

## Conclusions

The results of this study provide evidence of a potent *in vitro* and *in vivo* anti-inflammatory activity associated with PDE4 inhibition, as shown by inhibition of LTB_4_ production induced by LPS in human blood and by the suppression of NF-kB activation with the associated production of inflammatory cytokines in mice topically treated using an inhalation system that closely mimics the administration of dry powders in humans.

## Data Availability Statement

The datasets generated for this study are available on request to the corresponding author.

## Ethics Statement

All animal experiments were approved by the intramural animal welfare committee for animal experimentation of Chiesi Farmaceutici under protocol number 0013415-P-25/07/2011 complying with the European Directive 2010/63 UE, Italian D.Lgs 26/2014.

## Author Contributions

Conception and design: FS, AS, FR, MC, and GV. Laboratory testing: FS, FR, CB, AA, and PR. Data collection: FS, FR, CB, AA, and PR. Data analysis and interpretation: FS, AS, FR, MC, GV, CB, and PR. Drafting of manuscript: FS, AS, and GV.

## Funding

AS was the recipient of a research grant from Chiesi Farmaceutici S.p.A., that had no role in the study design, data collection and analysis, decision to publish, or preparation of the manuscript.

## Conflict of Interest

FS, FR, AA, MC, and GV are employees of Chiesi Farmaceutici S.p.A., which supported the research work.

The remaining authors declare that the research was conducted in the absence of any commercial or financial relationships that could be construed as a potential conflict of interest.

## Abbreviations

BLI, bioluminescence imaging; BALFs, bronchoalveolar lavage fluids; PDE4, phosphodiesterase 4; LTB_4_, leukotriene B_4_; LPS: lipopolysaccharide.

## References

[B1] Abramovitz M. Wong E. Cox M. E. Richardson C. D. Li C. Vickers P. J. (1993). 5-Lipoxygenase-activating protein stimulates the utilization of arachidonic acid by 5-lipoxygenase. Eur. J. Biochem. 215, 105–111. 10.1111/j.1432-1033.1993.tb18012.x 8344271

[B2] Ansaldi D. Hod E. A. Stellari F. Kim J. B. Lim E. Roskey M. (2011). Imaging pulmonary NF-kappaB activation and therapeutic effects of MLN120B and TDZD-8. PloS One 6, 3–10. 10.1371/journal.pone.0025093 PMC3178604 21966423

[B3] Armani E. Amari G. Rizzi A. De Fanti R. Ghidini E. Capaldi C. (2014). Novel class of benzoic acid ester derivatives as potent PDE4 inhibitors for inhaled administration in the treatment of respiratory diseases. J. Med. Chem. 57, 793–816. 10.1021/jm401549m 24400806

[B4] Biernacki W. A. Kharitonov S. A. Barnes P. J. (2003). Increased leukotriene B4 and 8-isoprostane in exhaled breath condensate of patients with exacerbations of COPD. Thorax 58, 294–298. 10.1136/thorax.58.4.294 12668789 PMC1746632

[B5] Bloemen P. G. van den Tweel M. C. Henricks P. Engels F. Kester M. H. de Loo P. G. (1997). Increased cAMP levels in stimulated neutrophils inhibit their adhesion to human bronchial epithelial cells. Am. J. Physiol. 272 (4), L580–L587. 10.1152/ajplung.1997.272.4.L580 9142928

[B6] Bonventre J. V. Huang Z. Taheri M. R. O’Leary E. Li E. Moskowitz M. A. (1997). Reduced fertility and postischaemic brain injury in mice deficient in cytosolic phospholipase A2. Nature 390, 622–625. 10.1038/37635 9403693

[B7] Brideau C. Van Staden C. Styhler A. Rodger I. W. Chan C. C. (1999). The effects of phosphodiesterase type 4 inhibitors on tumour necrosis factor-alpha and leukotriene B4 in a novel human whole blood assay. Br. J. Pharmacol. 126, 979–988. 10.1038/sj.bjp.0702387 10193778 PMC1571215

[B8] Caramori G. Romagnoli M. Casolari P. Bellettato C. Casoni G. Boschetto P. (2003). Nuclear localisation of p65 in sputum macrophages but not in sputum neutrophils during COPD exacerbations. Thorax 58, 348–351. 10.1136/thorax.58.4.348 12668802 PMC1746629

[B9] Diot P. Guillon A. Montharu J. Guillemain J. Schubnel V. Vecellio L. (2012). Pulmonary delivery of dry powders to rats: Tolerability limits of an intra-tracheal administration model. Int. J. Pharm. 434, 481–487. 10.1016/j.ijpharm.2012.05.013 22609125

[B10] Dixon R. A. F. Diehl R. E. Opas E. Rands E. Vickers P. J. Evans J. F. (1990). Requirement of a 5-lipoxygenase-activating protein for leukotriene synthesis. Nature 343, 282–284. 10.1038/343282a0 2300173

[B11] Drost E. M. Skwarski K. M. Sauleda J. Soler N. Roca J. Agusti A. (2005). Oxidative stress and airway inflammation in severe exacerbations of COPD. Thorax 60, 293–300. 10.1136/thx.2004.027946 15790984 PMC1747355

[B12] Edwards M. R. Bartlett N. W. Clarke D. Birrell M. Belvisi M. Johnston S. L. (2009). Targeting the NF-kappaB pathway in asthma and chronic obstructive pulmonary disease. Pharmacol. Ther. 121, 1–13. 10.1016/j.pharmthera.2008.09.003 18950657 PMC7172981

[B13] Giembycz M. A. Field S. K. (2010). Roflumilast: first phosphodiesterase 4 inhibitor approved for treatment of COPD. Drug Design Dev. Ther. 4, 147–158. 10.2147/DDDT.S7667 PMC2915539 20689641

[B14] Gonsalves C. S. Kalra V. K. (2010). Hypoxia-mediated expression of 5-lipoxygenase-activating protein involves HIF-1 and NF- B and MicroRNAs 135a and 199a-5p. J. Immunol. 184, 3878–3888. 10.4049/jimmunol.0902594 20194722

[B15] Guide for the Care and Use of Laboratory Animals (1996). Washington, D.C.: National Academy Press.

[B16] Gupta S. (2012). Side-effects of roflumilast. Lancet. 379 (9817), 710–711. 10.1016/S0140-6736(12)60304-3 22364756

[B17] Hill A. T. Campbell E. J. Hill S. L. Bayley D. L. Stockley R. A. (2000). Association between airway bacterial load and markers of airway inflammation in patients with stable chronic bronchitis. Am. J. Med. 109, 288–295. 10.1016/S0002-9343(00)00507-6 10996579

[B18] Hoover R. L. Karnovsky M. J. Austen K. F. Corey E. J. Lewis R. A. (1984). Leukotriene B4 action on endothelium mediates augmented neutrophil/endothelial adhesion. Proc. Natl. Acad. Sci. 81, 2191–2193. 10.1073/pnas.81.7.2191 6326110 PMC345463

[B19] Houslay M. D. (2010). Underpinning compartmentalised cAMP signalling through targeted cAMP breakdown. Trends Biochem. Sci. 35, 91–100. 10.1016/j.tibs.2009.09.007 19864144

[B20] Kim N. D. Chou R. C. Seung E. Tager A. M. Luster A. D. (2006). A unique requirement for the leukotriene B 4 receptor BLT1 for neutrophil recruitment in inflammatory arthritis. J. Exp. Med. 203 (4), 829–835. 10.1084/jem.20052349 16567386 PMC2118298

[B21] Mariotti F. Govoni M. Lucci G. Santoro D. Nandeuil M. A. (2018). Safety, tolerability, and pharmacokinetics of single and repeat ascending doses of CHF6001, a novel inhaled phosphodiesterase-4 inhibitor: two randomized trials in healthy volunteers. Int. J. Chronic Obstruct. Pulmonary Dis. 13, 3399–3410. 10.2147/COPD.S174156 PMC6203112 30425469

[B22] Moretto N. Caruso P. Bosco R. Marchini G. Pastore F. Armani E. (2015). CHF6001 I: a novel highly potent and selective phosphodiesterase 4 inhibitor with robust anti-inflammatory activity and suitable for topical pulmonary administration. J. Pharmacol. Exp. Ther. 352, 559–567. 10.1124/jpet.114.220541 25576075

[B23] Nassini R. Pedretti P. Moretto N. Fusi C. Carnini C. Facchinetti F. (2012). Transient receptor potential ankyrin 1 channel localized to non-neuronal airway cells promotes non-neurogenic inflammation. PloS One 7, e42454. 10.1371/journal.pone.0042454 22905134 PMC3419223

[B24] Oh H. J. Hwang D. W. Youn H. Lee D. S. (2013). In vivo bioluminescence reporter gene imaging for the activation of neuronal differentiation induced by the neuronal activator neurogenin 1 (Ngn1) in neuronal precursor cells. Eur. J. Nuclear Med. Mol. Imaging 40, 1607–1617. 10.1007/s00259-013-2457-0 23754760

[B25] Page C. P. Spina D. (2011). “Phosphodiesterase inhibitors in the treatment of inflammatory diseases,” in Handbook of Experimental Pharmacology. (Berlin Heidelberg: Springer-Verlag). 10.1007/978-3-642-17969-3_17 21695650

[B26] Peters-Golden M. (2009). Putting on the brakes: cyclic AMP as a multipronged controller of macrophage function. Sci. Signaling 2 (75), pe37. 10.1126/scisignal.275pe37 19531801

[B27] Profita M. Di Giorgi R. Sala A. Bonanno A. Riccobono L. Mirabella F. (2005). Muscarinic receptors, leukotriene B4 production and neutrophilic inflammation in COPD patients. Allergy: Eur. J. Allergy Clin. Immunol. 60 (11), 1361–1369. 10.1111/j.1398-9995.2005.00892.x 16197467

[B28] Rahman A. Fazal F. (2011). Blocking NF- B: an inflammatory issue. Proc. Am. Thoracic Soc. 8, 497–503. 10.1513/pats.201101-009MW PMC3359076 22052926

[B29] Sanchez-Galan E. Gomez-Hernandez A. Vidal C. Martin-Ventura J. L. Blanco-Colio L. M. Munoz-Garcia B. (2009). Leukotriene B4 enhances the activity of nuclear factor-kB pathway through BLT1 and BLT2 receptors in atherosclerosis. Cardiovasc. Res. 81, 216–225. 10.1093/cvr/cvn277 18852255

[B30] Schuliga M. (2015). NF-kappaB Signaling in Chronic Inflammatory Airway Disease. Biomolecules 5, 1266–1283. 10.3390/biom5031266 26131974 PMC4598751

[B31] Serezani C. H. Lewis C. Jancar S. Peters-Golden M. (2011). Leukotriene B4 amplifies NF-κB activation in mouse macrophages by reducing SOCS1 inhibition of MyD88 expression. J. Clin. Invest. 121, 671–682. 10.1172/JCI43302 21206089 PMC3026722

[B32] Soler-Cataluña J. J. Martínez-García M. Román Sánchez P. Salcedo E. Navarro M. Ochando R. (2005). Severe acute exacerbations and mortality in patients with chronic obstructive pulmonary disease. Thorax. 60 (11), 925–931. 10.1136/thx.2005.040527 16055622 PMC1747235

[B33] Stellari F. Bergamini G. Sandri A. Donofrio G. Sorio C. Ruscitti F. (2015a). In vivo imaging of the lung inflammatory response to Pseudomonas aeruginosa and its modulation by azithromycin. J. Trans. Med. 13, 251. 10.1186/s12967-015-0615-9 PMC4522964 26239109

[B34] Stellari F. F. Franceschi V. Capocefalo A. Ronchei M. Facchinetti F. Villetti G. (2012). In vivo imaging of transiently transgenized mice with a bovine interleukin 8 (CXCL8) promoter/luciferase reporter construct. PloS One 7, 1–9. 10.1371/journal.pone.0039716 PMC3386280 22761878

[B35] Stellari F. F. Lavrentiadou S. Ruscitti F. Jacca S. Franceschi V. Civelli M. (2014a). Enlightened Mannhemia haemolytica lung inflammation in bovinized mice. Vet. Res. 45, 1–6. 10.1186/1297-9716-45-8 24460618 PMC3906860

[B36] Stellari F. F. Ruscitti F. Pompilio D. Ravanetti F. Tebaldi G. Macchi F. (2017). Heterologous matrix metalloproteinase gene promoter activity allows in vivo real-time imaging of bleomycin-induced lung fibrosis in transiently transgenized mice. Front. Immunol. 8, 199. 10.3389/fimmu.2017.00199 28298912 PMC5331072

[B37] Stellari F. F. Sala A. Donofrio G. Ruscitti F. Caruso P. Topini T. M. (2014b). Azithromycin inhibits nuclear factor-κB activation during lung inflammation: an in vivo imaging study. Pharmacol. Res. Perspect. 2, 1–12. 10.1002/prp2.58 PMC4186419 25505605

[B38] Stellari F. Sala A. Ruscitti F. Carnini C. Mirandola P. Vitale M. (2015b). Monitoring inflammation and airway remodeling by fluorescence molecular tomography in a chronic asthma model. J. Transl. Med. 13, 336. 10.1186/s12967-015-0696-5 26496719 PMC4619338

[B39] Tsantikos E. Lau M. Castelino C. M. N. Maxwell M. J. Passey S. L. Hansen M. J. (2018). Granulocyte-CSF links destructive inflammation and comorbidities in obstructive lung disease. J. Clin. Invest. 128, 2406–2418. 10.1172/JCI98224 29708507 PMC5983324

[B40] Villetti G. Carnini C. Battipaglia L. Preynat L. Bolzoni P. T. Bassani F. (2015). CHF6001 II: a novel phosphodiesterase 4 inhibitor, suitable for topical pulmonary administration–in vivo preclinical pharmacology profile defines a potent anti-inflammatory compound with a wide therapeutic window. J. Pharmacol. Exp. Ther. 352, 568–578. 10.1124/jpet.114.220558 25576073

[B41] White A. J. Gompertz S. Stockley R. A. (2003). Chronic obstructive pulmonary disease. 6: the aetiology of exacerbations of chronic obstructive pulmonary disease. Thorax 58, 73–80. 10.1088/1751-8113/44/8/085201 12511727 PMC1746462

[B42] Wu K. Meyers C. A. Bennett J. A. King M. A. Meyer E. M. Hughes J. A. (2004). Polyethylenimine-mediated NGF gene delivery protects transected septal cholinergic neurons. Brain Res. 1008, 284–287. 10.1016/j.brainres.2004.02.051 15145767

[B43] Zarini S. Gijon M. A. Ransome A. E. Murphy R. C. Sala A. Gijón M. A. (2009). Transcellular biosynthesis of cysteinyl leukotrienes in vivo during mouse peritoneal inflammation. Proc. Natl. Acad. Sci. U.S.A. 106, 8296–8301. [pii] 10.1073/pnas.0903851106 19416808 PMC2688893

[B44] Zhou X. Gao X.-P. Fan J. Liu Q. Anwar K. N. Frey R. S. (2005). LPS activation of Toll-like receptor 4 signals CD11b/CD18 expression in neutrophils. Am. J. Physiol.-Lung Cell. Mol. Physiol. 288, L655–L662. 10.1152/ajplung.00327.2004 15563689

